# AI‐Driven Personalized Nutrition: Integrating Omics, Ethics, and Digital Health

**DOI:** 10.1002/mnfr.70293

**Published:** 2025-10-29

**Authors:** Celin Mundt, Büşra Yusufoğlu, Daniel Kudenko, Kerem Mertoğlu, Tuba Esatbeyoglu

**Affiliations:** ^1^ Department of Molecular Food Chemistry and Food Development Institute of Food and One Health Gottfried Wilhelm Leibniz University Hannover Hannover Germany; ^2^ Department of Chemistry Faculty of Science and Letters Istanbul Technical University Istanbul Türkiye; ^3^ L3S Research Center Leibniz Universität Hannover Hannover Germany; ^4^ Department of Horticulture Faculty of Agriculture Usak University Uşak Türkiye

**Keywords:** deep learning, machine learning, omics technologies, predictive health models

## Abstract

Personalized nutrition (PN) aims to prevent and manage chronic diseases by providing individualized dietary guidance based on genetic, metabolic, and lifestyle data. Artificial intelligence (AI) has become a key enabler in PN by analyzing large‐scale, multiomics datasets in obesity, diabetes, cardiovascular, and gastrointestinal disorders, where digital twins and health knowledge graphs support personalized interventions. Current findings demonstrate that AI models can guide microbiome‐based dietary interventions, and support obesity management, thereby extending the scope of conventional nutritional strategies as supported by deepened bibliometric analyses. This study highlights the global increase in AI‐based PN studies, accelerated by digital health demands and the COVID‐19 pandemic, and the expansion of traditional nutrition strategies through machine learning approaches with the integration of microbiome‐based models and omics. However, challenges such as algorithmic bias, limited generalizability, and data privacy remain. To overcome these issues, diverse datasets, explainable AI approaches, and standardized multicenter validation protocols are proposed. These steps are critical for transforming AI‐supported PN from a conceptual potential into a fair, reliable, and clinically applicable structure. The growing consensus in the literature highlights that AI can support individual and societal health goals by transforming nutrition science through predictive, adaptive, and ethically based approaches.

## Graphical abstract

This graphical abstract illustrates the concept of personalized nutrition, incorporating biological, dietary, and lifestyle factors. Artificial intelligence (AI)‐powered data analysis, digital tools and personalized nutrition recommendations target optimizing health and preventing diseases.

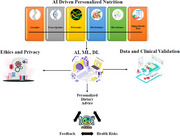

## 1 Introduction

Artificial intelligence (AI) is generally defined as a set of algorithms or systems designed to mimic some aspects of human intelligence. A more comprehensive definition of AI can encompass sophisticated systems that analyze data, reason, make informed decisions, and adapt those decisions to achieve complex goals. These systems operate in both physical and digital environments and respond dynamically to feedback to achieve their intended goals [1]. In recent years, AI applications have emerged in many areas, such as medicine, engineering, quality control, food, nutrition, basic sciences, and pharmaceuticals. The most important of these is the concept of “personalized nutrition” (PN), which has recently become critical for public health [2, 3, 4].

The National Institutes of Health (NIH) announced its 10‐year strategic nutrition plan to promote public health and prevent or address diseases and conditions affected by proper nutrition [5]. Proper nutrition plays a crucial role in reducing the incidence and progression of many chronic diseases, including cardiovascular disease, hypertension, stroke, Type 2 diabetes, metabolic syndrome, and certain types of cancer [6, 7]. PN not only helps prevent these diseases but also aids in identifying their root causes by considering individual variability. This concept is particularly valuable, as it addresses personal responses to dietary components on the basis of genotype, phenotype, behavior, personality, and sociopsychological and environmental factors [2, 8].

PN aims to provide tailored dietary recommendations for optimal health on the basis of an individual's specific profile, such as genetics, lifestyle, and health goals, unlike generic dietary guidelines. This approach recognizes the biological variability among individuals and is built on the understanding that people metabolize and respond differently to nutrients [9, 10]. Text Box 1 lists some of the widely used and less accessible tools validated for clinical use (Text Box 1). Recent clinical applications have demonstrated the feasibility of AI‐based nutritional guidance. For example, systems integrating continuous glucose monitoring (CGM) with AI‐driven algorithms have been successfully used to tailor carbohydrate intake for patients with Type 2 diabetes, resulting in improved glycemic control and reduced postprandial fluctuations [11, 12]. AI‐powered mobile applications now offer real‐time personalized dietary feedback by integrating multimodal data, including dietary logs, physical activity, CGM, and gut microbiome profiles. For example, a recent AI‐enabled nutrition program delivered via a mobile app significantly increased microbiome diversity and reduced waist circumference in a 6‐week intervention among healthy adults [13]. Additionally, large randomized trials, such as the ZOE METHOD study, have demonstrated that personalized AI‐driven dietary advice, informed by data on postprandial responses, the microbiome, and health history, produced greater improvements in cardiometabolic markers than standard dietary counseling [14].

To fully comprehend how dietary components affect human physiology, holistic analytical approaches known as nutriomics must be applied. These approaches integrate multiple biological layers to account for complex food–body interactions. By combining various omics platforms, a more comprehensive understanding of how specific food components modulate health through molecular and metabolic pathways can be obtained [15].

Omic biomarkers are a current approach that uses cutting edge technologies for practical applications to determine health status for PN strategies. These biomarkers cover a wide range of biological processes, including carbohydrate and fat metabolism, inflammation, oxidative stress, and microbiota‐derived metabolites [16]. Metabolomic analyses of circulating branched‐chain amino acids (isoleucine, leucine, and valine) and aromatic amino acids (tyrosine and phenylalanine) have been shown to predict future Type 2 diabetes risk in population‐based cohorts [17]. Mass spectrometry‐based lipidomic profiling has revealed specific phosphatidylcholine and sphingomyelin species associated with cardiovascular disease risk over a 10‐year follow‐up, outperforming traditional lipid markers [18]. Furthermore, circulating short‐chain fatty acids, such as acetate, propionate, and butyrate, have been linked to whole‐body insulin sensitivity and metabolic regulation, suggesting a role in obesity and inflammatory metabolic dysfunction [19].

**Text Box 1 Accessibility of different tools for personalization of nutrition [20]**

**Table:** 

Widely accessible tools	Less accessible tools
■ Demographic information (age, sex, and life stage) ‐ Phenotypic information ‐ Anthropometrics ‐ Standard clinical biomarkers (cholesterol, blood glucose, blood pressure, etc.) ‐ Biomarkers of nutrient levels	■ Genotypic and omics‐based information and tools ‐ Genetic testing and counseling ‐ Omics testing, including transcriptomics, proteomics, metabolomics, microbiomics, and xenomicrobiomics
■ Lifestyle information and tools ‐ Personal goals ‐ Physical activity ‐ Environment ‐ (Cultural) Preferences ‐ Smartphone applications (e.g., for diet tracking and planning) ‐ Wearable devices ‐ Dietary intake assessments	■ Lifestyle information and tools ‐ Sensors for energy intake ‐ Prepared or portioned meal delivery ‐ Fitness testing and exercise training ‐ Challenge testing ‐ Metabolism (oral‐glucose‐tolerance tests, mixed macronutrient challenge tests) ‐ Other systems, such as immune system and gut microbiota

Omics technologies are critical tools that strengthen the scientific foundation of PN. Metabolomic studies indicate how branched‐chain amino acids and short‐chain fatty acids can be used as biomarkers to predict the risk of Type 2 diabetes. Lipidomic profiles associate specific phospholipid and sphingomyelin species with cardiovascular diseases, while microbiome analyses hold potential for predicting individual responses to prebiotic and probiotic interventions. The aim of this review is not only to describe the existing literature on AI‐assisted PN but also to provide a unique critical analysis. By focusing particularly on explainable AI, federated learning (FL), ethical challenges, and real‐world implementation barriers, this work distinguishes itself from earlier reviews that primarily offered general frameworks. In doing so, our study synthesizes current trends in the scientific literature and offers guiding recommendations for future research.

**FIGURE 1:**
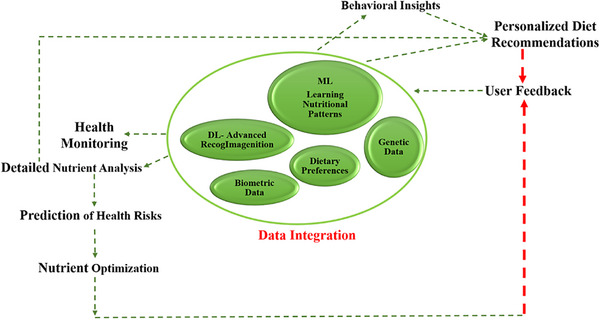
PN concept in terms of AI, ML, and DL (modified from [22, 23, 24]). AI, artificial intelligence; DL, deep learning; ML, machine learning; PN, personalized nutrition.

The terms machine learning (ML) and deep learning (DL), which are components of AI, are also integral to this context. Since AI and ML focus on improving performance with large and complex datasets, they can be used to analyze omics‐based measurements and construct predictive algorithms for nutritional outcomes [21]. However, beyond their technical definitions, recent literature emphasizes emerging challenges such as the need for explainable AI to increase transparency [33], the development of FL to protect user privacy [56, 57], and the difficulty of translating algorithmic insights into real‐world nutritional workflows due to interoperability and validation barriers [29, 30]. These aspects distinguish current research trends from earlier descriptive studies and represent critical frontiers in PN. AI, ML, and DL are often used interchangeably to describe intelligent systems or software. As illustrated in Figure 1, DL is a subdomain of ML, which itself is a subset of AI. AI generally integrates human‐like learning and reasoning into systems, ML emphasizes model construction through data learning, and DL uses multilayer neural networks to extract hierarchical patterns. “Deep” refers to the number of processing layers through which data passes. ML and DL are key technologies enabling intelligent decision‐making and automation, making AI more effective in health‐related domains. These systems are closely linked to data science, as they help extract meaningful insights and contribute to intelligent, domain‐specific solutions [22].

As visualized in Figure 1, data integration in AI‐assisted PN involves ML and DL algorithms that combine genetic data, biometric measurements, and individual dietary preferences to address personal nutritional needs. Personalized dietary recommendations are then generated based on these inputs, and the system improves iteratively with user feedback. This adaptive feedback mechanism enhances system accuracy over time. Key components that strengthen the functionality of this model include health monitoring, nutritional analysis, health risk prediction, behavioral profiling, and personalized guidance on the basis of integrated data streams.

This literature review explores the concept of “personalized nutrition” by highlighting the opportunities enabled by AI. However, while AI introduces significant advances, it also presents concerns regarding users and their interaction with the technology. These include factors such as lifestyle, phenotypic and genotypic variability, biomarker accessibility, data privacy, ethical implications, algorithmic bias, technology availability, healthcare professionals’ awareness, and the ability of both developers and end‐users to interpret and implement AI‐based recommendations accurately. Bibliometric analysis has revealed distinct research clusters, such as microbiome–nutrition interactions, ethical frameworks, and digital health interventions that directly reflect the thematic gaps emphasized in our critical synthesis. By incorporating these trends into the discussion, we connect global research trajectories with the challenges and opportunities identified in this review. This review aims to critically synthesize current developments in AI‐assisted PN by integrating omics data, ethical frameworks, and digital health innovations. Although previous studies have mostly provided a descriptive overview, the originality of this work stems from its focus on emerging approaches such as explainable AI, FL, and real‐world implementation barriers. Moreover, this review combines narrative synthesis with bibliometric evaluation to provide a comprehensive and critical perspective that highlights both opportunities and limitations, thereby aiming to guide future research and applications.

## 2 Big Data Analytics and Predictive Modeling in Personalized Nutrition Strategies

Recent technological advancements have enabled individuals to continuously monitor various aspects of their health and lifestyle through digital tools [25]. E‐health platforms and wearable technologies have revolutionized real‐time health data collection, offering a cost‐effective and dynamic approach to personalized health profiling [26]. These tools generate vast amounts of user‐specific data that, when processed through advanced analytical models, can be translated into meaningful and actionable dietary insights. In practice, multimodal data streams, dietary logs, physical activity, sleep, heart rate variability, and CGM are already being combined with machine‐learning pipelines to personalize advice; ML models trained on CGM, microbiome, and clinical features can predict postprandial glycemic responses and guide individualized meal selection [11, 12]; while an 18‐week app‐based personalized program has been shown to improve several cardiometabolic markers versus general advice [14].

PN offers an evidence‐based alternative to generalized dietary recommendations by integrating individual genetic, phenotypic, clinical, and nutritional data. Compared with standardized interventions, this tailored approach is more likely to meet individual health needs and improve dietary adherence [27, 28]. However, although current nutrition practices are moving toward personalization, prevention, and participation, the predictive potential of AI models remains limited in the absence of robust, stratified nutritional datasets. Addressing this gap requires the development of case‐specific databases and the validation of intervention outcomes through blinded, randomized trials. Additionally, real‐world deployments should report standard model performance metrics, discrimination area under the receiver operating characteristic / Precision ‐ recall area under curve (AUROC/PR‐AUC), calibration (calibration plots, Brier score) and include external validation and subgroup analyses to ensure transportability and fairness. Recent work in clinical nutrition modeling has shown that acceptable discrimination can coexist with calibration differences across subpopulations, underscoring the need to report both [29, 30].

The integration of ML into PN is pivotal in transforming data‐driven insights into predictive tools. ML algorithms can support clinicians in prioritizing interventions based on an individual's pathophysiological profile, thereby increasing decision‐making through biologically relevant reasoning [31, 32].

A personalized nutritional approach accounts for interindividual variability in metabolism, genetics, microbiome composition, and biochemical responses. Within this framework, AI contributes to elucidating the molecular mechanisms underlying nutrient–health interactions by identifying biomarkers linked to specific dietary interventions and health outcomes [33]. The process begins with the collection of individual input data, such as demographics, health status, dietary habits, physical activity levels, and medical history, which are then processed through AI‐based algorithms to deliver tailored nutritional recommendations. These systems may also incorporate food classification and dietary behavior tracking to refine and adjust interventions over time [4]. Image‐based diet tracking supported by AI (Keenoa system) has shown moderate‐to‐strong relative validity against reference methods in healthy adults and people living with diabetes, illustrating the feasibility of longitudinal, low‐burden data capture in PN workflows [34].

Beyond the clinical setting, AI‐driven nutrition technologies have broader implications for the food industry and public health. Tools for automated food recognition and nutritional estimation enable consumers to better monitor intake, support behavior modification, and make more informed food choices. As AI continues to evolve, its integration into nutrition science holds great promise not only in enhancing health outcomes but also in shaping responsible innovation and ethical application [4]. To maximize the impact on public health, datasets should be harmonized (common ontologies/metadata), pipelines should be interoperable across sources (wearables, electronic health records (EHRs), microbiomes, and metabolomics), and evaluations should routinely include drift monitoring and recalibration procedures in addition to accuracy summaries.

The ability of AI to process complex and voluminous datasets makes it a powerful tool for developing real‐time predictive models and intelligent decision‐making systems for nutrition [35]. Nonetheless, one of the primary challenges remains the accurate metabolic characterization of individuals, which is influenced by numerous internal and external factors, including genetic background, gut microbiota, metabolomic profiles, diet, and physical activity [16, 36].

**FIGURE 2:**
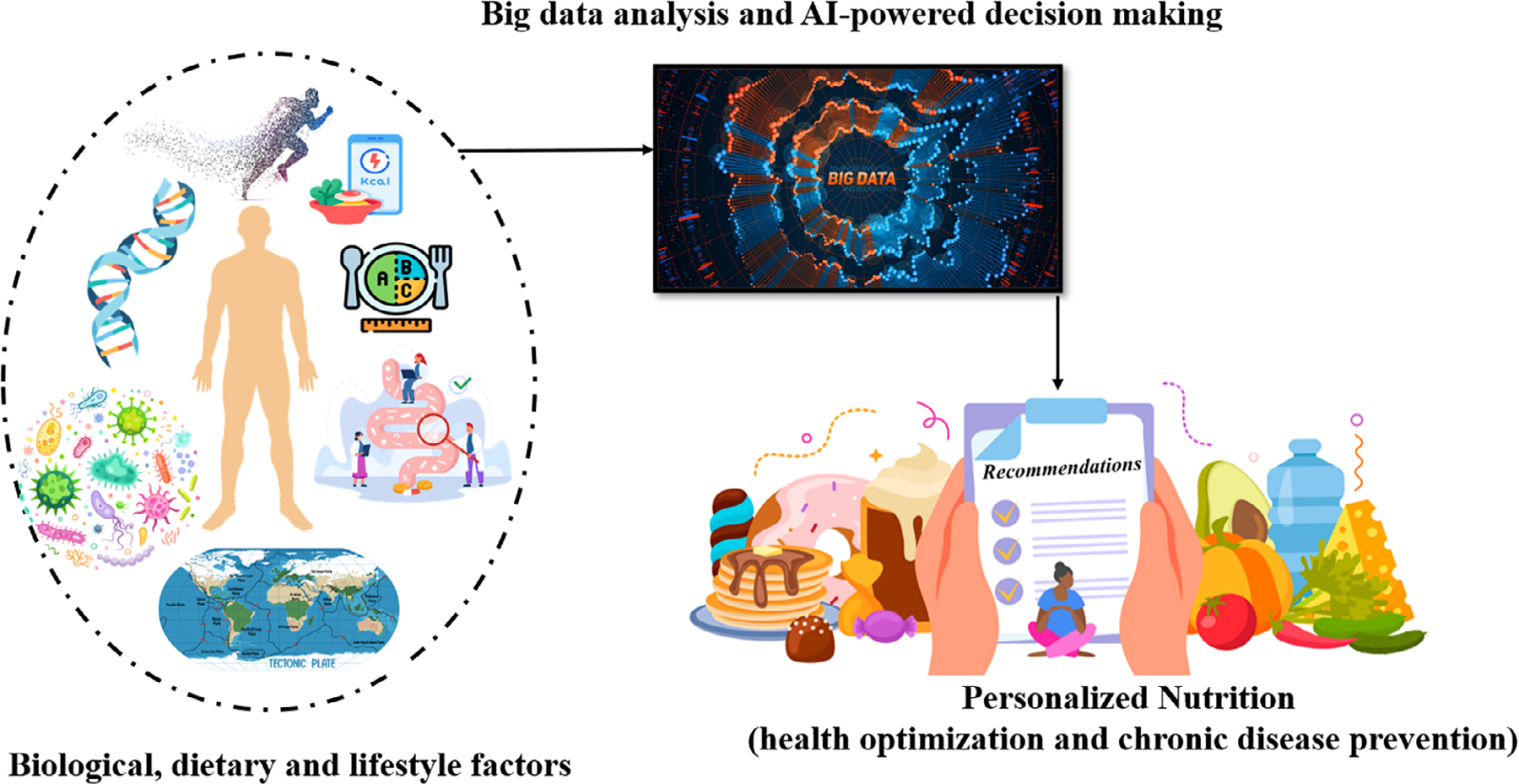
The concept of PN on the basis of deep phenotyping, big data analysis and AI‐powered decision‐making (modified from [42, 43]). AI, artificial intelligence; PN, personalized nutrition.

As illustrated in Figure 2, integrating data from various sources, such as demographic profiles, genomic and omics‐based data, and lifestyle metrics, enables the development of precise nutrition strategies tailored to individual health needs. This process, referred to as deep phenotyping, includes the evaluation of observable traits that result from a combination of genetic, epigenetic, environmental, and behavioral influences [37, 38]. Nutrition plays a fundamental role in modulating both physical and mental health, exerting effects on hormonal regulation, the immune response, neurotransmitter balance, and the microbiota–gut–brain axis. These systems are vital for reducing inflammation, managing stress, and preserving cognitive performance [28].

Importantly, the relationship between nutrition and health is bidirectional, while a balanced diet promotes physiological homeostasis, poor dietary habits contribute to the development of noncommunicable diseases (NCDs), such as obesity, Type 2 diabetes, cardiovascular diseases, respiratory disorders, and various cancers [39, 40]. Among these, NCDs represent a critical global challenge, accounting for approximately 71% of all deaths worldwide. Consequently, the integration of AI‐based decision support systems with large‐scale data holds great potential for reducing NCD‐related mortality and enhancing public health through personalized, data‐driven nutritional strategies [41].

Ensuring the reliability of AI‐based PN approaches, reporting model accuracy metrics is of critical importance. In this context, studies frequently employ measures such as area under the curve (AUC), PR‐AUC, Brier scores, and calibration curves. Beyond statistical accuracy, external validation and cross‐validation with independent cohorts are indispensable for assessing clinical applicability. Real‐world implementations provide substantial evidence in this regard. The ZOE program demonstrated the capacity to predict postprandial glycemic responses by leveraging microbiome and clinical features, while the Keenoa application confirmed the feasibility of image‐based diet tracking in populations with diabetes. Integration of CGM devices with machine‐learning algorithms has further strengthened the effectiveness of personalized nutritional interventions. Collectively, these cases indicate that AI‐supported models are increasingly moving from theoretical constructs to practical applications in real‐world contexts.

## 3 Artificial Intelligence as a Catalyst in the Evolution of Personalized Nutrition

“Personalized nutrition” has emerged as a transformative paradigm in public health by empowering individuals with tailored dietary tools and strategies aligned with their unique physiological and behavioral profiles [8]. Ongoing investment in digital health technologies and computational models is expected to catalyze further advances that were once considered speculative, thereby translating precision‐based dietary interventions from theoretical frameworks into clinical and practical realities [44].

Recent studies have underscored the capacity of AI, a computational field that simulates human cognitive processes, to enhance PN applications. Specifically, AI‐driven systems have been shown to improve dietary quality by integrating data from dietary questionnaires and indices such as the Healthy Eating Index and the Mediterranean Diet Score, which allows for the quantifiable monitoring of dietary patterns [45]. In addition, randomized controlled trials have demonstrated measurable health benefits: Bermingham et al. [14] reported significant improvements in cardiometabolic markers (LDL cholesterol and HbA1c) with AI‐personalized diets, whereas Popp et al. [46] reported greater weight loss in individuals following a personalized diet than following a standard low‐fat diet. Moreover, evidence suggests that AI‐personalized diets may induce distinct changes in the composition and diversity of the gut microbiota, reflecting metabolic adaptations not captured by conventional methods [13].

AI‐based tools leverage ML and DL approaches to increase the precision of dietary assessment through automated food recognition and portion estimation. ML refers to algorithms that learn patterns from data, whereas DL, a subfield of ML, uses layered neural networks to process high‐dimensional information with minimal human intervention. Applications such as “Keenoa” demonstrate the practical utility of these systems. This mobile platform combines AI‐powered image recognition with user feedback to track food intake and estimate nutrient content with higher accuracy than self‐reported methods do, particularly in pediatric and adolescent populations where recall bias is prominent [47, 48, 49].

Furthermore, AI enables the identification of gaps and inefficiencies in traditional nutrition care models by analyzing large‐scale, heterogeneous health datasets. Recent advancements include electronic nose (E‐nose) systems, devices that mimic olfactory perception via AI algorithms, and electronic tongue (E‐tongue) systems, which integrate optical and electrochemical sensors to analyze the compositional quality of foods and beverages [50, 51]. These technologies, when integrated with standard analytical chemistry, have significantly improved the precision of food quality assessments [52, 53]. Despite these advances, a major challenge remains in the clinical validation of AI‐based dietary intake assessments. Validation with nutritional experts and medical professionals is essential to ensure that outputs generated by AI systems are both accurate and clinically reliable [39, 40]. Without such validation, there is a risk that algorithmic predictions may overlook context‐specific dietary behaviors or misclassify nutrient intakes, potentially compromising patient care.

Advanced computational models, including artificial neural networks, decision trees, and transformer‐based architectures such as OpenAI's generative pretrained transformer (GPT‐4), have expanded the reach of AI across both clinical and industrial domains. For example, [54] demonstrated that AI/ML tools utilizing the Anaconda platform could predict nutrition‐related outcomes on the basis of inputs such as age, sex, body mass index (BMI), and biochemical parameters, including cholesterol, glucose, and thyroid hormones.

As summarized in Table 1, these algorithms are increasingly applied in areas such as food fraud detection, disease risk stratification, and nutrition education. In one study, Bayesian network models accurately identified food fraud scenarios with a 63.8% success rate and traced points of adulteration within the supply chain with 71.3% accuracy [55]. Similarly, E‐nose systems combined with ML achieved 95.8% accuracy in classifying the purity of olive oil and fruit juice samples, outperforming traditional quality control methods [52, 53].

**TABLE 1:** Recent PN and food studies by means of AI algorithms.

Model/device	Target	Summary	Key limitations/clinical applicability	Reference
CNN, BiLSTM, NLP	Identifying dietary patterns: traditional and staple, communal and festive, and westernized and convenience‐oriented	Predicting obesity and dyslipidemia patterns and accuracy↑ Public health strategies ↑	Limited generalizability, requires large datasets.	[60]
SHAP model based on ML	Predict biomarkers for myasthenia gravis	Microbiota‐metabolite integrated myasthenia gravis	Limited availability for diet‐specific validation; not applicable to dietary practice.	[61]
MLR, RFR, and DT	Predicting food energy content and classifying food items	Predicting and categorizing ↑ Health risks ↓ (Accuracy values MLR; 0.99, RFR; 0.987DT; 1.00)	Laboratory‐based validation; accuracy may be reduced with mixed meals; clinical relevance is limited.	[62]
ANN, SVM, RF, elastic net models based on ML	Predicting the early termination of nutrition therapy	Optimize the management of nutrition support team, responsibilities people ↓, Predicting ↑ANN sensitivity ↑	Data are limited to hospital groups only; external validation is lacking.	[63]
Deep generative AI model and ChatGPT/AI model diet plan	AI‐based nutrition recommendation	Effectiveness of ChatGPT ↑ Meal variety ↑ Accuracy ↑ Generalization ↑	Nutrient amounts are often imprecise; there is no clinical validation.	[40, 64]
OpenAI's GPT‐4 model	Assessment of the exercise prescriptions of user experience	Accessibility in terms of cost ↓	Not targeted nutrition; limited clinical trials.	[65]
ChatGPT (version 4.0)	AI‐based diet plans	Quality of inputs ↑ applicability of diet plans ↑	Cannot separate nutrition values correctly; requires human control.	[59]
RF based on ML	Estimating of breakfast postprandial glucose responses	Feasibility of continuous glucose monitoring and postprandial glucose responses ↑	Limited range; not approved for clinical studies.	[66]
DTx	DTx for people with T2D	Glucose monitoring (CGM) ↑ T2D management↑	Cost is high; patients need to integrate with digital tools.	[67, 68, 69]
ML algorithms/chatbot and wearable EEG	Development of personalized medicine for MDD	Cognitive assessment ↑Multiomics approach ↑	Psychiatric application; some ethical concerns.	[70]
Stance4Health Nutritional APP	The Python 3.7 module, the libraries of pandas, SciPy, seaborn, matplotlib	Nutritional education to the users ↑	Average clinical results; orientation required.	[71]
DT, ML/DT, DT, DL, NN, and extended short‐term memory models	DT technology to predict postprandial glycemic responses	AI‐enhanced devices ↑ DT‐supported PN interventions↑	Difficulty in collaborative studies; clinical studies are limited.	[67]
FL	FL is able to achieve a high‐performing global model	Local user data can be protected↑ while the server's computing and storage burdens ↓	Assessment is limited; there is no PN applications.	[56]
SMC	Ensures data privacy through encryption	SMC models in FL computational overhead↑ smaller ↓	Industry applications are available; there is no clinical PN workflows.	[56]
AI	Identifying “a novel PN” approach	AI‐based dish nutrition evaluation ↑ proper food choices ↑	Early concept stage; clinical validations are lacking.	[72]
Electronic tongues	Designed liquid analysis, optical, electrical, gravimetric sensors, biosensors	Specialized multisensory ↑	Industry‐focused; clinical PN application is limited.	[50, 51]
ChatGPT with LLM and RS model	Meal plan for NCDs using	Needed nutrition experts for appropriateness	Requires expert validation; needs cultural orientation	[73]
AI‐ML Anaconda/Python	Dietary guidelines	Mathematical modeling ↑	Technical feasibility; there is not tested clinically.	[54]
AI‐powered personalized meal plans	Establishing healthy eating habits	Individual's health conditions ↑ Preferences AI meal plan ↑	Commercial applications are limited.	[74]
Web‐based diabetes care by AI	Diabetes self‐management	Managing diabetes ↑	Digital divide barriers need continuous engagement.	[58]
AI4Food‐NutritionFW	Study introduces “AI4Food‐NutritionFW”	Evaluating eating behavior's ↑	Initial stage; not validated in clinical studies.	[26]
BN	Identification of the type of food fraud and the point of adulteration in the supply chain	Identification food fraud ↑	Accuracy averages; indirect PN relevance.	[55]
E‐nose system—ML algorithm (SVM, KNN, and DT)	Identification of olive oil food fraud and adulteration	SVM ↑	Datasets are limited to selected oil types; limited clinical PN studies.	[52]
AI based PROTEIN advisor app (PN for healthy living)	Protein app uses a modified Technology Acceptance Model (mTAM)	Healthier living ↑	Limited studies are initial‐stage.	[75]
E‐nose system—ANN	Identifying the distinctions between pure and industrial varieties of juice	Total accuracy of 95.8% ↑	Food industry context; no PN applications yet.	[53]
PCA SVM ANN CNN RF models	AI‐enhanced devices like electronic noses and tongues	Product development ↑	High and big data requirements; targeted proof‐of‐concept.	[76]
FL	Is it possible to train models without the need to transfer data to a central location?	Increased training and prediction processes ↑	Statistical heterogeneity, high cost, significant limitations in clinical applications.	[57]
Decision tree classification algorithm	Classification of civet coffee and noncivet coffee	Classification of coffee ↑	Only food classification; no clinical PN applicatiosn.	[77]

AI, artificial intelligence; ANN, artificial neural networks; BiLSTM, Bidirectional Long‐Short Term Memory; BN, Bayesian network; ChatGPT, Generative Pre‐Trained Transformer; CNN, convolutional neural network; DL, deep learning; DT, decision tree; DTx, digital twin; EEG, electroencephalography; FL, federated learning; LLM, large language model; MDD, major depressive disorder; ML, machine learning; MLR, multiple linear regression; NCD, non‐communicable disease; NLP, natural language processing; PCA, principal component analysis; RF, rainforest; RS, recommender system; SHAP, SHapley Additive exPlanations; SMC, secure multi‐party computation; SVM, support vector machine; T2D, Type 2 diabetes.

With increasing concerns about digital ethics and user privacy, secure computing models such as FL and secure multiparty computation (SMC) have gained prominence. FL enables decentralized model training without sharing raw data, whereas SMC allows multiple parties to compute functions over private data without revealing them to others [56, 57]. These technologies are essential for ensuring that AI‐based nutritional tools remain compliant with ethical standards and privacy regulations.

In addition to back‐end analytics, AI has begun to influence front‐end nutritional behavior changes. Conversational agents such as ChatGPT and web‐based platforms have shown promising results in enhancing health literacy and encouraging healthier choices through dynamic, personalized interactions [58, 59]. Importantly, rather than claiming that AI enhances understanding of human nutrition, it is more accurate to state that AI facilitates the understanding of complex interactions among diet, physiological phenomena, eating behavior, and environmental factors [2, 20]. This framing reflects a cautious but scientifically grounded view, emphasizing AI's role as an enabler rather than a categorical paradigm shift. Through advanced sensing technologies (E‐nose, E‐tongue), privacy‐preserving algorithms (FL, SMC), and interactive systems (ChatGPT), PN is progressing toward a more intelligent, responsive, and ethically grounded model of care. As such, AI serves not only as a technological tool but also as a catalyst in the evolutionary trajectory of PN and public health.

AI should therefore be regarded not as a revolutionary replacement for nutrition science but as a supportive instrument that enhances the understanding of diet–environment interactions. Although these technologies provide valuable contributions by extracting meaningful patterns from large‐scale datasets, current tools also face notable limitations. Language models such as ChatGPT, for instance, offer motivational advantages in user interaction but remain insufficient in calculating precise nutrient quantities, which limits their clinical reliability. Many AI applications also lack validation across diverse populations, resulting in restricted generalizability. Hence, the role of AI in PN must be addressed with balanced consideration, recognizing both the opportunities and the current constraints.

## 4 Critical Challenges Hindering the Advancement of AI in Personalized Dietetics

Although the integration of AI into PN offers transformative potential, it also introduces a complex array of challenges that must be addressed to ensure ethical, equitable, and effective implementation [4]. Concerns about the potential replacement of healthcare professionals and questions regarding liability in the case of algorithmic errors are among the most prominent issues raised by clinicians and regulatory bodies [78, 79]. Concrete examples illustrate these risks: for instance, commercial nutrition‐tracking applications have experienced data breaches that exposed thousands of users’ sensitive health information [84], while algorithmic misclassification of dietary patterns from underrepresented populations has been documented, highlighting how nonrepresentative datasets can reinforce existing disparities [81]. International regulatory frameworks, such as the European Union's General Data Protection Regulation (GDPR) and the United States’ Health Insurance Portability and Accountability Act (HIPAA), have been highlighted as foundational references for ensuring data privacy and accountability in AI‐driven PN. In addition, ethical guidelines and expert recommendations stress algorithmic transparency, explainability, and human oversight as prerequisites to prevent overreliance on automated decision‐making systems [33, 80]. These concerns highlight the need for clearly defined accountability structures and robust regulatory frameworks before AI systems can be fully integrated into healthcare practices.

A second major challenge concerns privacy and data security, as AI applications in PN rely heavily on the continuous collection and analysis of personal health data. The sensitivity of such information raises significant ethical concerns, particularly when considering the risks associated with data breaches or unauthorized third‐party access [20]. Moreover, algorithmic bias stemming from nonrepresentative training datasets can result in unequal outcomes across different ethnic, cultural, or socioeconomic groups, reinforcing existing health disparities [4, 81]. To mitigate these risks, regulatory and academic recommendations increasingly emphasize algorithmic auditing methods, including preprocessing (data balancing), in‐processing (bias correction), and postprocessing (outcome equalization), as essential safeguards against unfair outcomes in health‐related AI [82, 85].

Another structural issue is the lack of generalizability of AI models due to the underrepresentation of diverse populations in nutritional datasets. Current models often reflect the characteristics of a narrow demographic, limiting their predictive power when applied to broader populations. To overcome this, rigorous validation of AI systems across heterogeneous groups is essential, alongside efforts to expand the diversity of training datasets [4]. Moreover, interdisciplinary collaboration between data scientists, nutritionists, ethicists, and clinicians is crucial for building AI tools that are both technically sound and contextually appropriate.

One of the inherent biological challenges in PN is the accurate metabolic characterization of individuals. This requires accounting for complex internal factors such as genetic polymorphisms, microbiota composition, and metabolomic profiles, as well as external influences such as diet, physical activity, and the environment [16, 36, 43]. Although omics technologies offer unprecedented insights into individual biology, the integration of these multidimensional datasets remains technically and economically demanding. Despite advances in ML and bioinformatics, the development of user‐friendly and cost‐effective PN protocols remains limited by these complexities [16, 86].

Consumer acceptance also plays a pivotal role in the success of AI‐driven PN tools. Factors such as perceived benefits, usability, trust in AI systems, and clarity in the communication of personalized recommendations determine user compliance and long‐term adherence [23]. One of the most significant barriers to adoption is the lack of transparency in how AI systems generate dietary advice. When users do not understand the basis of algorithmic decisions, their willingness to engage with these tools diminishes regardless of the accuracy of the recommendations. Detopoulou et al. and Gerlich. indicated how public trust is strongly tied to regulatory clarity and AI literacy, reinforcing the need for transparent communication and educational efforts to accompany technological deployment [33, 87].

Technological limitations further complicate food classification processes, which are foundational for dietary assessment systems. Accurate identification of food items is influenced by factors such as preparation methods, lighting conditions, image quality, and the presence of mixed ingredients. DL models require large, well‐annotated datasets to train food recognition systems effectively. Techniques such as transfer learning, fine‐tuning, and data augmentation can improve model performance, but substantial infrastructure and expertise are needed to implement these solutions [49]. Moreover, the opacity of AI technologies, which are often perceived as “black boxes,” hinders public understanding and informed decision‐making. As these systems typically rely on proprietary algorithms, users are often unaware of how outputs are generated [88]. In a recent study, higher levels of AI literacy were positively correlated with acceptance and trust, suggesting that education and transparency are critical for successful integration [87].

From an ethical standpoint, it is essential to ensure that AI systems treat individuals fairly and without bias, regardless of gender, race, or ethnicity. This can be achieved through algorithmic auditing methods, including preprocessing (data balancing), in‐processing (bias correction), and postprocessing (outcome equalization) techniques [89]. When applied systematically, these strategies can mitigate unfair outcomes and foster greater public trust in AI systems used for health purposes. From legal and social perspectives, integrating AI into nutritional science raises questions about liability in cases of harm, equity of access to advanced digital tools, and the distribution of risks and benefits across society. Future policies and frameworks must therefore strike a balance between innovation and accountability to ensure that AI enhances healthcare without exacerbating inequalities or eroding patient trust [33, 82, 90].

Technical challenges are further compounded by operational issues, including inconsistent data quality, software bugs, inadequate supervision, and physician overreliance on AI outputs. Even well‐developed AI systems may produce suboptimal results if the users, whether patients or healthcare providers, lack sufficient understanding of the system's functionality or limitations [79, 82]. Additionally, economic barriers such as the cost of wearables, limited access to real‐time data infrastructures, and insufficient integration with electronic health records hinder the widespread adoption of PN technologies [8, 10, 83]. Although AI and omics technologies hold great promise for reshaping PN, the complexity of human metabolism, technological limitations, and ethical concerns remain significant hurdles. Bridging this gap requires not only advancements in computational modeling and sensor technologies but also robust ethical frameworks that prioritize fairness, data privacy, and inclusive design. Only then can AI evolve from a powerful analytical tool to a truly transformative force in nutritional science and public health.

A review of the literature indicates how omics technologies (genomics, transcriptomics, proteomics, metabolomics, and microbiomics) provide the scientific foundation of PN approaches. These technologies enable multilayered assessment of genetic architecture, metabolic capacity, and microbial ecosystems, allowing dietary interventions to be tailored to individual biological differences. Single‐nucleotide polymorphisms (SNPs) at the genomic level serve as predictors of obesity or Type 2 diabetes risk, while metabolomic profiles involving short‐chain fatty acids (SCFAs) or branched‐chain amino acids directly reflect diet–metabolism interactions. Microbiome‐based data further support the identification of individuals more likely to benefit from probiotic or prebiotic interventions. The contribution of omics technologies in PN extends beyond the description of biological diversity; integration with AI and ML algorithms facilitates the development of predictive models. Multiomics‐driven approaches clarify interindividual differences in glycemic responses or diet‐induced inflammation, moving beyond standardized dietary guidelines toward dynamic and individualized recommendations. Despite this potential, omics‐based PN faces several limitations, including high costs, lack of data standardization, biological variability, and ethical or privacy concerns. Clinical translation requires multicenter validation studies, regulatory compliance, and explainable frameworks within AI‐based decision support systems. In conclusion, omics technologies strengthen the scientific infrastructure of PN, and their integration into clinical decision‐making will represent a critical success factor for the future of this field.

## 5 Privacy, Bias, and Fairness of AI

The rapid integration of AI into healthcare and PN has created transformative opportunities, yet it simultaneously raises complex challenges concerning privacy, fairness, and algorithmic bias. As ML and big data technologies increasingly rely on sensitive personal information, safeguarding against unauthorized access and unintentional data exposure has become a major concern [84]. In addition to direct data breaches, AI can extract personal details such as emotional states, ethnic identities, or political preferences from seemingly insensitive indicators such as activity logs or location data, intensifying public unease about surveillance and autonomy. In this context, privacy issues extend beyond technical risks to include both deontological concerns, such as perceived loss of control, and consequentialist outcomes, for instance, insurance discrimination or employment limitations resulting from data misuse [85]. The rising demand for health monitoring apps and AI‐powered tools has led to widespread data collection, intensifying the need for robust privacy protocols. The example of China's social scoring system illustrates how AI can be leveraged to evaluate and rank individuals, thereby limiting access to services on the basis of algorithmic categorization [84].

Although legal frameworks such as the HIPAA and the GDPR were established to protect user data, they are often inadequate in the face of increasingly complex AI systems. To address ethical dimensions, global efforts such as the UNESCO Global Initiative on the Ethics of Autonomous and Intelligent Systems advocate transparency and fairness in digital systems [85]. However, even advanced techniques such as pseudonymization or anonymization may fail to fully safeguard privacy, prompting a shift toward network‐based solutions that transfer learned models rather than raw data to reduce exposure risk during data sharing [78, 79].

Bias in AI systems represents another critical challenge. It can arise at various stages, from data collection and model development to analysis, and may lead to the systematic exclusion or misrepresentation of specific groups [80, 85]. In the context of PN, biased data can result in inaccurate recommendations or the marginalization of underrepresented populations identified several forms of bias, including sampling, representation, confirmation, and generative biases, can impact the fairness of AI outcomes [81]. Personalization specifically refers to the adaptation of dietary recommendations to an individual's genetic, metabolic, and lifestyle data, in agreement with the findings of Adams et al. [20]. From a privacy perspective, this personalization process raises concerns because it requires the continuous collection and storage of sensitive health information, creating risks of misuse or unauthorized disclosure [33]. From a biased perspective, personalization may inadvertently exacerbate inequalities when training datasets underrepresent certain ethnic, cultural, or socioeconomic groups, leading to systematically less accurate recommendations for those populations [2].

Addressing such bias requires both technical interventions and ethical oversight. Techniques such as resampling, preprocessing, oversampling, and synthetic data generation are employed to correct imbalances and reduce discriminatory outputs [81]. Nevertheless, ensuring fairness extends beyond algorithm design and requires deliberate choices in model selection, with emphasis on the equality of outcomes across diverse user groups. Otherwise, systems may perpetuate harmful stereotypes or reinforce structural inequalities.

**FIGURE 3:**
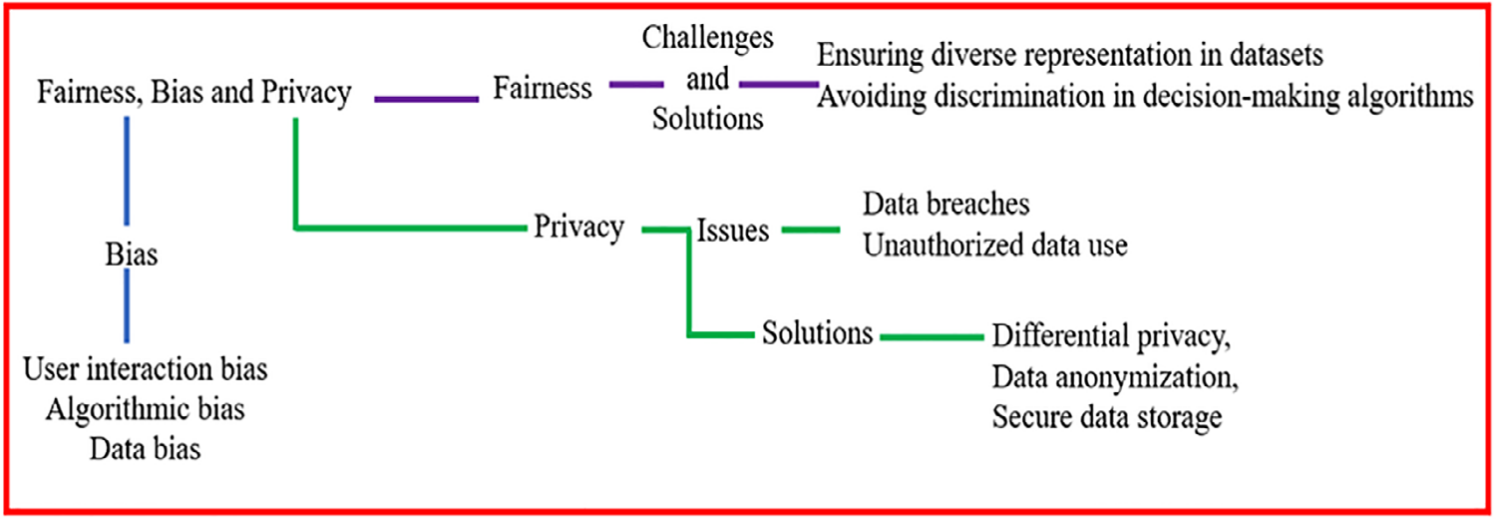
Relationships among privacy, bias and fairness in AI. AI, artificial intelligence.

The interplay between privacy, bias, and fairness is a determinant of AI system quality, as represented in Figure 3. Building resilient models not only improves interpretability and adaptability, such as via transfer learning, but also strengthens resistance to misuse and adversarial attacks [91]. However, technical improvements alone are insufficient if users can not trust or understand how AI decisions are made.

The public perception of AI remains limited, partly due to the opacity of proprietary systems and the abstract nature of algorithmic processes. This complexity hinders the informed evaluation of potential benefits and risks [88]. Recent findings suggest that familiarity with AI fosters greater acceptance, whereas trust significantly shapes attitudes and behavioral intentions [87]. Another persistent challenge in PN is the metabolic characterization of individuals, which requires consideration of genetic, microbiomic, and environmental factors [16, 36, 43]. Although omics and AI technologies offer unprecedented insights, their clinical translation is still hindered by economic and technical constraints. The complexity of metabolic regulation, combined with insufficient stratified data, limits the generalizability and scalability of AI solutions in nutrition [86].

Further complicating implementation is the issue of user acceptance. Variables such as the design of personalized services, clarity of recommendations, and perceived benefits all influence adherence to AI‐generated dietary guidance. Most critically, the lack of transparency in algorithmic processes remains a barrier to trust [23].

From a systems perspective, challenges also arise in food classification, which is an essential task for dietary assessment. The accuracy of classification impacts the precision of nutrient analysis, and it is heavily influenced by factors such as preparation methods, lighting, and image quality. DL models require extensive, well‐labeled datasets, and their performance can be improved with techniques such as fine‐tuning, data augmentation, and transfer learning [49].

Despite these issues, AI should not be seen as a threat to healthcare professionals. Instead, it is intended as a decision‐support system that enhances clinical outcomes without replacing human judgment. As noted by [82, 90, 92], clinicians are expected to retain authority in decision‐making and intervene when algorithmic ambiguity arises. AI is not meant to override physician expertise but rather to increase the speed, accuracy, and timeliness of healthcare delivery [93]. Nevertheless, the concept of technological singularity, where AI surpasses human intelligence, raises questions about the long‐term role of healthcare professionals [94]. Although this scenario remains speculative, professionals must prepare to adapt, acquire the skills necessary to operate AI systems, and critically interpret their outputs [95]. In the near term, AI is more likely to function as a powerful assistant than a replacement, increasing the effectiveness of health practitioners while supporting data‐informed, individualized interventions [93].

Although current AI applications in PN demonstrate considerable potential, their long‐term success depends on addressing key challenges of data privacy, algorithmic bias, fairness, and transparency. Limited representation of ethnic minorities and cultural diversity in training datasets reduces predictive accuracy and risks reinforcing health disparities, as seen in the misclassification of dietary items from Asian and Latin American cuisines. Data security concerns are equally critical, with breaches in commercial nutrition‐tracking apps exposing thousands of users’ sensitive health information and undermining trust. Regulatory frameworks such as the GDPR in the European Union, emphasizing transparency and explicit consent, and HIPAA in the United States, mandating strict rules for secure storage and sharing of health records, provide essential safeguards. Yet compliance with these standards must be complemented by robust data protection mechanisms, ethical inclusivity, and cultural representation. The success of AI‐driven PN will ultimately depend not only on algorithmic precision but also on its ability to safeguard user rights and ensure equitable participation across diverse populations.

## 6 Opportunities and Current Research Frontiers in AI‐Driven Dietary Interventions and Health Optimization

In the evolving paradigm of PN, AI stands out as a pivotal technology for translating complex biological and behavioral data into actionable, individualized dietary strategies. Recent studies combining AI, omics sciences, and behavioral analytics have revealed the immense potential of this integration to address the increasing burden of nutrition‐related chronic diseases through precision health interventions. These developments not only present concrete achievements but also unveil prospective opportunities for advancing predictive, preventative, and participatory models of nutritional care.

A cornerstone study in the field, the Food4Me trial, which was conducted across seven European countries, demonstrated that PN advice on the basis of individual characteristics outperformed conventional, generalized dietary guidance in improving nutritional behavior. This trial confirmed the feasibility of delivering tailored nutrition via digital platforms and highlighted the scalability of AI‐supported approaches in population‐wide interventions. These findings suggest that future public health strategies can benefit from AI‐enhanced models that adapt evidence‐based recommendations to individual lifestyles, genotypes, and phenotypes, particularly in resource‐limited or remote contexts. In this context, patients with obesity, Type 2 diabetes, cardiometabolic syndromes, and gastrointestinal disorders represent priority populations, as AI‐driven personalization has shown measurable benefits in weight management, glycemic control, cardiovascular risk reduction, and gut microbiome modulation [14, 67, 72].

Building on this, generative AI models such as ChatGPT have been evaluated for their ability to replicate or enhance rule‐based algorithms. In a comparative study using data from 20 obese individuals, ChatGPT successfully generated personalized dietary suggestions; however, it displayed notable limitations in quantitative precision regarding macro‐ and micronutrient distributions. Despite these shortcomings, the model's capacity to deliver motivational dialogue and contextual reasoning signals an emerging opportunity to embed conversational AI into digital nutrition platforms, thereby increasing patient engagement and adherence. Nevertheless, concerns remain about cultural adaptation and potential biases: if generative AI is trained primarily on Western‐centric data, it may fail to capture local dietary patterns, religious food restrictions, or region‐specific nutritional risks, leading to inequities in recommendations [33, 81]. Future improvements in numerical processing and clinical validation may transform these models into decision‐support tools within dietetics.

AI integration has also been extended to digital twin technologies and personal health knowledge graphs (PHKGs), offering a paradigm shift toward real‐time, adaptive disease management. A proposed framework for diabetes care demonstrated the utility of combining PHKGs with biometric and lifestyle data to generate continuously updated, patient‐specific care plans. Such systems hold the promise of redefining chronic disease management by allowing for dynamic, multifactorial interventions. Clinical trials have shown how digital twins enable PN and improve metabolic outcomes in patients with Type 2 diabetes and metabolic dysfunction‐associated fatty liver disease [67, 68]. Future expansions of this framework may incorporate behavioral feedback loops, environmental exposures, and socioeconomic variables, paving the way for context‐aware, holistic nutrition systems.

In the domain of gastrointestinal disorders, AI‐assisted PN has demonstrated therapeutic efficacy. A study involving IBS‐M patients revealed that individualized, AI‐guided diets significantly outperformed standard protocols, as evidenced by symptom reduction and improved microbiota profiles. These findings underscore that gastrointestinal disorders constitute another high‐need population where AI‐driven nutrition can provide substantial benefits by linking microbiome analysis with personalized dietary interventions [13]. This emphasizes the opportunity to use AI not only for symptom tracking and diet personalization but also for identifying biomarkers predictive of dietary responsiveness in patients with functional GI disorders.

Moreover, algorithmic models such as decision trees have been used to stratify dietary recommendations on the basis of phenotypic and behavioral traits. In a longitudinal study by [8], AI‐informed interventions led to meaningful improvements in nutrient intake quality. This affirms the opportunity to refine traditional nutritional counseling using predictive models that adapt to individual variability in metabolic response, dietary patterns, and behavioral change readiness. For example, predictive algorithms have demonstrated strong translational potential in diabetes prevention programs and obesity interventions, showing that patients at higher cardiometabolic risk can particularly benefit from adaptive dietary feedback [14, 96]. Furthermore, integrating psychosocial variables into such models remains an underexplored avenue with high translational potential.

A more advanced model was employed in the 18‐week randomized controlled trial by [14], where AI‐generated food scores incorporating microbiome data, cardiovascular biomarkers, and glycemic response resulted in improved compliance and metabolic outcomes relative to standard care. These results illuminate the opportunity to operationalize complex biomarker data into user‐centric formats such as food scoring, potentially enhancing health literacy and self‐management. Such approaches appear especially valuable in cardiometabolic disease populations, where traditional dietary counseling often faces adherence challenges [97]. Broadening these models to include emerging biomarkers such as metabolomic signatures or stress hormones could further augment personalization.

Public attitudes toward AI in healthcare have been systematically assessed by [97] and [98], revealing both optimism and ethical apprehension. Although concerns persist about clinician replacement and algorithmic opacity, there is growing acknowledgment that AI can serve as an adjunct rather than a replacement. This reflects an opportunity to develop hybrid models of care where clinicians retain oversight but benefit from AI‐derived insights, particularly in data‐heavy domains such as PN. To address these concerns, codesign processes involving patients from diverse cultural and socioeconomic backgrounds are essential to mitigate bias and improve trust in AI‐assisted nutrition systems [33, 81]. Education and codesign approaches involving both practitioners and patients will be critical to fostering trust and effective implementation. AI is expected to transform the nutritional landscape beyond recommendation systems. As projected by [96], applications may extend to early risk detection, automated dietary diagnostics, dynamic health trajectory modeling, and appointment triage. Each of these domains represents a fertile opportunity to integrate nutritional interventions into broader healthcare infrastructures, particularly through electronic health records and telehealth platforms.

The convergence of AI with omics technologies, including genomics, proteomics, and metabolomics, offers a deep phenotyping framework for dietary precision. Laying ML onto these data enables the identification of key molecular pathways influencing nutrient metabolism and disease susceptibility. This creates opportunities to develop mechanistically informed dietary interventions that move beyond phenotype‐based targeting. In practice, this integration could be particularly transformative in oncology, cardiometabolic disease prevention, and diabetes care, where omics‐based biomarkers can help identify at‐risk individuals and guide dietary interventions tailored to disease progression [5, 16, 42]. Future directions may include the development of AI tools that simulate nutrient–gene interactions or predict long‐term health outcomes from short‐term dietary patterns via systems biology models.

## 7 Evolving Trends and Conceptual Mapping in AI‐PN to Days Highlighted by Bibliometric Evidence

Bibliometric evidence has been employed to explore the evolving directions of AI‐driven PN, thereby highlighting gaps in the existing literature and underscoring the originality of this review. The analysis revealed distinct research clusters, such as microbiome–nutrition interactions, ethical frameworks, and digital health interventions that directly correspond to the thematic gaps emphasized in our critical synthesis. By incorporating these trends into the discussion, this review situates global research trajectories within the challenges and opportunities identified throughout the study.

**FIGURE 4:**
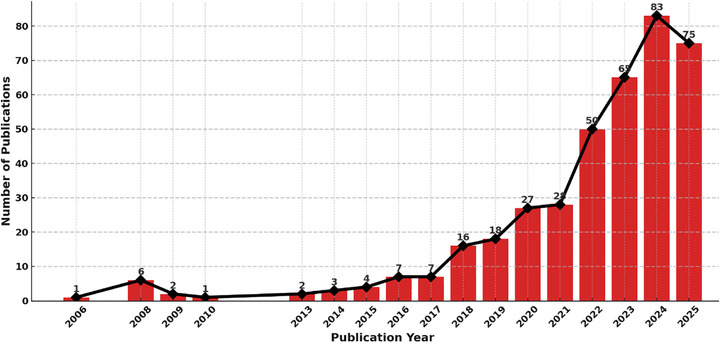
Annual distribution of publications on AI‐driven personalized nutrition. AI, artificial intelligence.

The annual distribution of publications on AI‐driven PN, illustrated in Figure 4, reveals a clear upward trajectory in scholarly output over the past two decades, reflecting the field's growing scientific relevance and technological feasibility. The earliest academic publication dates back to 2006. From 2006 through 2015, research activity remained sparse and sporadic, with fewer than 10 publications per year. This early phase likely represents foundational explorations into the integration of AI within the field of nutritional sciences, preceding the broader adoption of precision health paradigms. Between 2006 and 2019, the number of publications remained sparse, likely reflecting both the technological limitations of the time, such as underdeveloped ML infrastructure and limited access to personal health data, and the emerging nature of PN itself as a scientific domain [99]. The purpose of presenting these bibliometric patterns is not only to demonstrate the historical trajectory of the field but also to provide an introductory framework that contextualizes the subsequent discussion. By highlighting how publication trends have evolved in parallel with technological innovation and public health challenges, this section establishes the rationale for examining current opportunities and research frontiers in AI‐driven PN.

A notable shift began approximately in 2020, aligning with significant advancements in AI, digital health tools, and the increasing affordability of biosensors and genomic technologies. This transition was further accelerated by the global impact of the COVID‐19 pandemic, which underscored the need for remote, personalized, and data‐driven healthcare solutions [100]. As a result, the number of studies rose sharply from 2021 onward, reaching a peak in 2024, with 83 articles, suggesting that the field had matured into a recognized interdisciplinary research area. Although the data for 2025 exhibit a modest numerical decline, this figure reflects only publications indexed in the Web of Science database up to June and therefore does not represent the entirety of the calendar year. Despite this temporal limitation, the current count of 75 publications has already approached the total output observed in 2024, underscoring the sustained momentum within the area. Thus, the bibliometric overview is not presented as an isolated descriptive exercise but rather as an entry point into broader conceptual mapping. This ensures that the reader understands how shifts in research output mirror the maturation of AI‐PN as a discipline and provides the foundation for interpreting the thematic clusters and scientific collaborations that follow.

**FIGURE 5:**
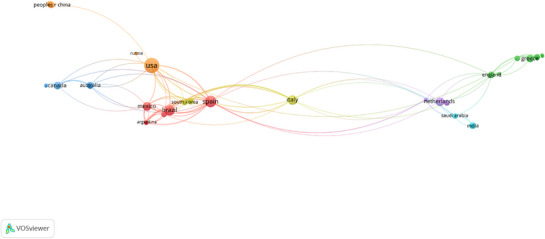
International collaboration network in AI‐driven PN research (from Voswiever). AI, artificial intelligence; PN, personalized nutrition.

Cluster‐based network visualization, produced via VOSviewer, illustrates the structure of international scientific collaboration in the field of PN (Figure 5). Each node represents a country, with the size indicating its overall publication volume and the color denoting its membership in a specific collaboration cluster. The links between nodes reflect the strength of coauthorship relationships, revealing patterns of geographical proximity, research intensity, and regional integration.

The United States appears to be the most central and influential node in terms of both publication volume and interconnectivity. As the primary hub within the orange cluster, it maintains strong bilateral ties with countries such as Brazil, Mexico, Russia, and China, indicating its role as a global leader and connector in this field. The USA's dominant position likely stems from its early adoption of omics‐based research, its investments in AI‐driven health technologies, and its participation in globally funded nutrition initiatives [101]. Several distinct clusters are evident across the network. The red cluster connects countries such as Spain, Brazil, Mexico, and Argentina, reflecting strong transatlantic collaboration, possibly influenced by shared linguistic or cultural ties. The blue cluster, which includes Canada and Australia, represents high‐income, English‐speaking nations that have established parallel research agendas with moderate connectivity to the USA. The green cluster, where Greece stands out, reflects a more localized European collaborative structure, likely shaped by EU research funding frameworks. Meanwhile, the purple cluster connects England, the Netherlands, and Saudi Arabia, pointing to Northern and Western European alliances with increasing outreach toward the Gulf region. Italy and Spain serve as bridges across clusters, facilitating integration between European, Latin American, and North American research efforts. These bridging nations play a critical role in enabling interdisciplinary and cross‐border knowledge exchange. By integrating these bibliometric insights into collaboration networks, this section provides a contextual foundation for readers to understand how geographic, cultural, and policy‐driven research agendas shape the development of AI‐PN. This framing ensures that subsequent thematic analyses are interpreted within the broader landscape of global scientific exchange.

**FIGURE 6:**
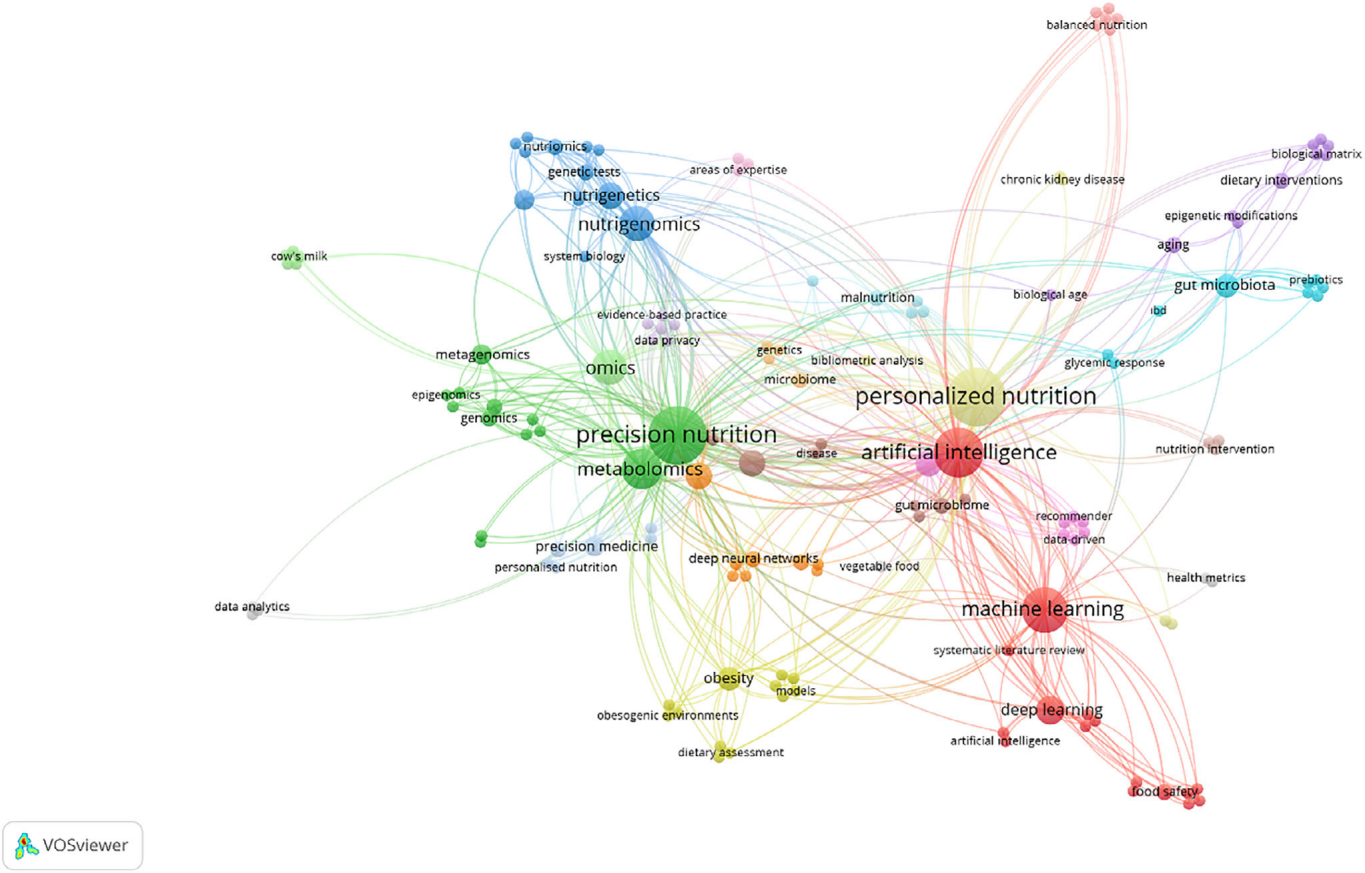
Keyword co‐occurrence network in AI‐driven PN (Voswiever). AI, artificial intelligence; PN, personalized nutrition.

VOSviewer analysis derived from the scientific literature on PN reveals the conceptual structure, interdisciplinary linkages, and thematic density of research in this evolving field (Figure 6). At the core of the network lies the term “personalized nutrition”, which serves as the dominant conceptual nucleus. Its high centrality and extensive linkages underscore its role as the thematic anchor that integrates diverse scientific disciplines. Notably, “artificial intelligence” and “machine learning” are positioned adjacent to PN, forming a dense subnetwork characterized by mutual interdependence. This proximity suggests that computational methodologies have become indispensable tools for managing the complexity inherent in personalized dietary recommendations.

Situated to the left of the central cluster, a densely interconnected green subnetwork comprises key terms such as “omics”, “genomics”, “metabolomics”, “epigenomics”, and “nutrigenomics”, reflecting the molecular and systems biology foundations of PN. The strong colinkage between omics and precision nutrition underscores the field's dependence on high‐dimensional biological data to stratify populations and develop individualized dietary plans. The positioning of “nutrigenetics” and “nutrigenomics” near the boundary between the green and blue clusters suggests their dual role in both data generation (omics) and clinical application (precision nutrition). Their connectivity to “system biology” and “genetic tests” also points to the mechanistic emphasis on understanding metabolic individuality at the genomic and proteomic levels.

In the lower‐right quadrant, “machine learning”, “deep learning”, and “deep neural networks” constitute a red cluster representing the computational backbone of AI‐based nutritional modeling. The dense interlinkages among these terms and their connections to “food safety”, “dietary assessment”, and “recommendation systems” reflect their growing utility in automated diagnostics, prediction of metabolic responses, and intelligent dietary decision support. This computational cluster's adjacency to “artificial intelligence” illustrates that ML and DL are not isolated techniques but rather operational engines of AI within this interdisciplinary framework.

Smaller clusters, such as purple (gut microbiota, prebiotics, and epigenetic modifications) and yellow (obesity and obesogenic environments) clusters, indicate specialized research niches. The linkage between the “gut microbiome” and “personalized nutrition” supports the hypothesis that microbial diversity plays a key role in dietary responsiveness, an area currently under active investigation. The colocation of “obesity” with “models” and “dietary assessment” suggests an applied focus on AI's predictive capacity in obesity prevention, particularly in understanding behavioral and metabolic determinants.

Terms such as “data privacy”, “evidence‐based practice”, and “systematic literature review” situated near the intersections of multiple clusters signal growing concerns over data governance, validation of AI outputs, and translational fidelity of AI‐generated nutritional recommendations. Their proximity to central nodes suggests that ethical, regulatory, and methodological considerations are not peripheral but rather integrated within the discourse of AI‐driven nutritional science. Furthermore, the peripheral placement of nodes such as “data analytics” and “cow's milk” suggests isolated or emerging lines of inquiry that are yet to be fully integrated into mainstream discourse. By explicitly linking these conceptual clusters to broader scientific questions, the section now highlights how bibliometric evidence maps the intellectual structure of the field and guides future research directions. The emphasis on areas such as gut microbiome diversity, obesity modeling, and computational decision support ensures that conceptual mapping serves as a bridge between historical publication trends and the opportunities for innovation discussed in later sections.

## 8 Future Directions of AI in Personalized Nutrition

The future of PN lies at the intersection of AI, multiomics technologies, and systems biology. As digital health systems evolve, AI offers unmatched capabilities to integrate and interpret vast, heterogeneous biological data to create dynamic, individualized dietary recommendations. However, the success of such systems depends not only on algorithmic power but also on the biological and methodological depth of the data they rely upon.

Nutrigenomics, computational biology, and multiomics technologies play critical roles in the development of personalized dietary interventions [10]. Although genomics allows for detailed mapping of the genome, including coding and noncoding regions, to identify genetic variants and disease susceptibilities, it is important to recognize the limitation that genes do not consistently produce direct biological outcomes, complicating predictions of nutritional responses [102]. In this context, nutriproteomics has emerged as a promising field, using proteomic techniques to study nutrient‒protein interactions that determine bioavailability and function. This systems‐level approach enhances our understanding of dietary responses, supports novel biomarker discovery, and strengthens the links between diet, metabolic pathways, and chronic disease development [9, 103]. These advances offer promising opportunities to inform AI‐driven models with high‐resolution molecular insight. Omics‐based approaches will represent one of the most critical success criteria for the future of PN; however, achieving this goal requires multicenter validation, standardization of data, and the establishment of ethical frameworks.

AI can serve as a powerful integrative tool in these contexts by connecting data layers from genomics, transcriptomics, proteomics, metabolomics, microbiomics, and even xenomicrobiomics with clinical, behavioral, and environmental factors. Assessment tools that underpin PN already incorporate diverse individual characteristics, such as age, sex, physiological status, dietary intake, lifestyle, and the gut microbiota, whereas AI provides a computational framework to synthesize these inputs and extract actionable patterns [16]. However, omics research presents specific challenges for AI integration. Variability among healthy and diseased populations, time‐dependent fluctuations in metabolite levels, and data heterogeneity make replication and generalization difficult [104]. In particular, metabolomics exemplifies a “highly dynamic dataset” because metabolite concentrations fluctuate rapidly in response to factors such as diet, circadian rhythms, medication use, and acute physiological changes, which complicates reproducibility and long‐term predictive modeling. Although internal validation within training datasets and external validation across populations are standard practices, true replication remains elusive in highly dynamic datasets such as metabolomics. This restricts the robustness of ML predictions unless appropriate quality control, normalization, and temporal modeling techniques are employed. Integrative omics approaches that analyze larger datasets may offer a partial solution by identifying consistent drivers of disease or nutritional response, although distinguishing causality from correlation must be addressed primarily through epidemiological associations and well‐designed human clinical studies rather than generic in vivo validation [9, 86, 105].

One promising area for future development is the use of adaptive AI models that are able to account for dynamic physiological and metabolic states. For example, integrating caloric load and caloric restriction, both of which are known to influence metabolic health and longevity [10], into personalized dietary algorithms could allow for more context‐aware interventions that align with circadian rhythms, hormonal cycles, or acute disease states. Nevertheless, the implementation of AI in PN is not without critical ethical, methodological, and technical challenges. Concerns include the risk of algorithmic bias, data privacy breaches, lack of model transparency, and underrepresentation of diverse populations in training datasets. For example, overly reductionist datasets may fail to account for cultural, socioeconomic, and genetic diversity, leading to inequitable recommendations or systemic bias in clinical outcomes.

To overcome these challenges, several measures can be adopted. First, AI model development must prioritize fairness and transparency, incorporating diverse training data and clearly defined decision‐making pathways. Recent proposals, such as explainable AI frameworks [33] and FL approaches [56, 57], provide concrete methodological avenues to ensure that predictive models remain both privacy‐preserving and interpretable. Moreover, real‐world deployments must report not only discrimination metrics but also calibration and subgroup analyses [29, 30], otherwise transportability to clinical practice remains limited. Second, standardization of omics data collection and processing is needed to enable reproducibility and model generalizability. Third, hybrid frameworks combining human expertise with AI‐generated insights should be developed to preserve clinical oversight while leveraging computational efficiency. Finally, educating both healthcare professionals and end‐users about the strengths and limitations of AI‐guided nutrition will be essential to promote trust and responsible adoption. Although significant progress has been made in applying AI to PN, future efforts must focus on integrating validated, high‐dimensional omics data; addressing methodological barriers in replication and causality; and ensuring ethical, inclusive, and clinically meaningful applications. With thoughtful advancements, AI‐driven nutrition can evolve from descriptive analytics to predictive, adaptive, and mechanistically informed systems that optimize human health on an individualized scale.

The future success of AI‐based PN will depend not only on technological innovation but also on resolving critical challenges such as data privacy, algorithmic bias, and fairness. Underrepresentation of ethnic and cultural diversity in training datasets risks amplifying bias, while data breaches may undermine user trust. For this reason, approaches aligned with regulatory frameworks such as GDPR and HIPAA must be inclusive, secure, and ethically robust. Furthermore, bibliometric evidence highlights the gut microbiome as a core research cluster, emphasizing its critical role in predicting postprandial responses and guiding microbiota‐targeted nutritional interventions. Ethical dimensions also emerge as a central cluster, underscoring the urgent need to address algorithmic bias, data protection, and equitable access. Collectively, these insights indicate that future PN research must integrate both biological complexity and ethical safeguards to achieve clinically relevant and socially responsible outcomes.

## 9 Conclusion

AI is rapidly redefining the landscape of PN by enabling the integration and interpretation of complex, multidimensional data ranging from genetic and metabolic profiles to behavioral and environmental factors. This review explores how AI technologies, including ML, DL, and decision‐support systems, are being leveraged to develop individualized dietary interventions with greater precision, scalability, and clinical relevance.

Through the incorporation of multiomics platforms, including genomics, metabolomics, proteomics, and microbiomics, AI systems are beginning to capture the intricate biological variability that underpins human nutritional responses. Tools such as digital twins, PHKGs, and real‐time biometric monitoring offer promising avenues for dynamic and adaptive nutrition platforms, which are capable of continuously tailoring recommendations in alignment with physiological changes, health goals, and environmental inputs. Despite these advancements, challenges remain. Issues such as algorithmic bias, data privacy, model interpretability, and underrepresentation of diverse populations must be addressed through robust validation frameworks, ethical standards, and inclusive research designs. Furthermore, the complexity of omics data requires improved causal inference methodologies and standardization across studies to enable meaningful integration into AI pipelines.

As the field continues to evolve, the future of AI in PN lies in its potential to shift dietary strategies from reactive to predictive, from generalized to individualized, and from static to context aware. As technologies mature, interdisciplinary collaboration among data scientists, clinicians, nutritionists, ethicists, and policymakers will be essential to harness AI responsibly and equitably.

In summary, AI represents both a technological tool and a conceptual framework for revolutionizing nutrition science. To ensure effective translation into real‐world practice, the following actionable recommendations can be drawn from this review:

For researchers: Prioritize the development of diverse and representative datasets, strengthen external validation across heterogeneous populations, and design explainable AI frameworks to improve transparency and reproducibility.

For policymakers: Establish clear AI validation guidelines, create robust ethical standards, and implement strong data governance protocols that safeguard privacy while promoting equitable access.

For industry stakeholders: Invest in explainable, user‐friendly, and interoperable AI‐driven platforms, with a focus on clinical applicability, consumer trust, and integration into healthcare and nutrition services.

By aligning scientific innovation with ethical and policy frameworks, AI can move from experimental promise to being a practical and trustworthy tool in global nutrition practice.

## Conflicts of Interest

The authors declare no conflicts of interest.

## Data Availability

No data were used for the research described in the article.
